# A POINT OF NO RETURN: A QUALITATIVE ANALYSIS ON RETURN MIGRATION AND AGING OF JAPANESE INDIVIDUALS LIVING IN NEW YORK

**DOI:** 10.1093/geroni/igad104.0993

**Published:** 2023-12-21

**Authors:** Itsuko Toyama, Taeko Nakashima

**Affiliations:** St. Andrew’s University, Osaka, Kyoto, Japan; Nihon Fukushi University, Mihama, Aichi, Japan

## Abstract

Our previous quantitative research studies have revealed that many older Japanese individuals living in overseas communities maintain hope of receiving culture-oriented care or harbor aspirations of returning to Japan to live out their final days. In this study, we analyzed the qualitative data of a free-answer question on anxieties about aging from a quantitative research study on older Japanese individuals living in Greater New York in 2018. We aimed to clarify the qualitative characteristics of anxieties among three groups: those who had decided to stay in the United States (“abiding”), those who had decided to return to Japan (“returning”), and those who had not yet decided (“undecided”). In the quantitative study, 2,057 questionnaires were distributed, with an overall return rate of 29.7%. The respondents were divided into three groups based on their answers regarding their intentions for the location of their final residence: the United States, Japan, or undecided. We performed the KJ method (affinity diagram) to visualize the differences and commonalities of their anxieties. The following is a summary of the findings: (1) Although most of the respondents are economically advantaged and healthy, “ambiguous“ anxieties about aging in New York arise in all three groups; (2) All three groups express uneasiness about supporting themselves after paying medical and nursing care costs; (3) Those in the “abiding” group express loneliness due to the societal relationship vulnerability in New York; (4) Those in the “returning” group worry about the additional burden of undergoing legal procedures and re-adjusting to the current Japanese society.

